# Beyond pruritus in Alagille syndrome: potential effects of maralixibat on fibrosis and portal hypertension–insights from two case studies

**DOI:** 10.3389/fmed.2025.1707258

**Published:** 2026-01-16

**Authors:** Jesús Quintero-Bernabeu, Cristina Padrós-Fornieles, Maria Mercadal-Hally, Mauricio Larrarte-King, Simone Mameli, Mar Miserachs-Barba, Lis Vidal-Valdivia, José Andrés Molino-Gahete, Antonio Moreno-Galdo

**Affiliations:** 1 Pediatric Hepatology and Liver Transplant Unit, Department of Pediatrics, Vall d’Hebron Barcelona Hospital Campus, ERN Rare Liver ERN TransplantChild, Barcelona, Spain; 2 Department of Pediatric Surgery, Vall d’Hebron Barcelona Hospital Campus, Barcelona, Spain; 3 Department of Pediatrics, Vall d’Hebron Barcelona Hospital Campus, Barcelona, Spain

**Keywords:** Alagille syndrome, cholestasis, Ileal Bile Acids Transporter inhibitors, liver fibrosis, portal hypertension, serum bile acids

## Abstract

**Introduction:**

Alagille syndrome (ALGS) is a genetic disorder primarily affecting the liver, often leading to cholestasis and pruritus. Ileal Bile Acid Transporter inhibitors (IBATi), such as maralixibat, have shown promise in controlling pruritus and potentially modifying disease progression.

**Case report:**

We report two patients with ALGS treated with maralixibat. Case 1 involved a 10-days-old newborn presenting with cholestasis, jaundice, and acholic stools. Genetic testing confirmed a pathogenic JAG1 mutation. Despite preserved liver function, the patient was evaluated for liver transplantation (LT) due to severe pruritus and portal hypertension. Maralixibat initiation led to marked improvement in pruritus, serum bile acids, bilirubin, and cholesterol. Liver biopsies performed 13 months apart demonstrated fibrosis regression from cirrhosis to stage F2. Elastography showed decreased liver stiffness, reduced splenomegaly, and improved platelet counts. Liver enzymes transiently increased, but treatment continued without dose adjustment. Case 2 involved a 15-years-old female with moderate portal hypertension and mild pruritus. After 24 months of maralixibat, pruritus resolved completely, serum bile acids decreased, liver stiffness and splenomegaly improved, and platelet counts increased. Liver enzymes remained mildly elevated without requiring treatment modification. These findings suggest maralixibat may improve portal hypertension and hepatic injury, even in patients with less severe biochemical abnormalities.

**Conclusion:**

In ALGS, maralixibat treatment improved pruritus, lowered serum bile acids, and suggested potential benefits on fibrosis and portal hypertension, indicating a possible role beyond symptomatic relief.

## Introduction

Alagille syndrome (ALGS) is an autosomal dominant disorder, mainly characterized by hepatic involvement with cholestasis and elevated γ- glutamyl transferase, alongside variable extrahepatic features (1). Two genes, JAGGED1 (JAG1) and NOTCH2, are implicated in ALGS, with pathogenic JAG1 variants or deletions found in 94.3% of patients meeting phenotypic criteria, while NOTCH2 mutations account for 2.5% of cases (2).

Hepatic involvement occurs in about 90% of ALGS cases and can be severe enough to require liver transplantation (LT). Around 60% of ALGS patients either die or need a LT before 18 years of age. The median age for transplantation is 2.8 years, with 72% of cases occurring within the first 5 years of life, primarily due to complications from persistent cholestasis (mainly intractable pruritus) and portal hypertension (1). Ileal Bile Acid Transporter inhibitors (IBATi) have been introduced to address cholestatic pruritus in patients with ALGS (3, 4). These medications have shown potential in controlling pruritus effectively, and early research hints at their ability to modify the natural progression of the disease (5). Although the hypothesis that reducing serum bile acids (sBA) levels may mitigate its toxic effect on the liver and potentially alter the progression of fibrosis/cirrhosis seems plausible, there is still a lack of solid data to support this. The aim of this study is to describe two patients with ALGS in whom treatment with the IBATi maralixibat was associated not only with a reduction in serum bile acid levels and pruritus, but also with an improvement in hepatic fibrosis.

## Case report

Case 1: a 10-days-old new born, the first child of non-consanguineous parents, was admitted with jaundice and pale stools. The infant was born full-term, weighing 2.6 kg, following an uneventful pregnancy with normal prenatal ultrasounds and no maternal infections. The family had no history of liver disease or miscarriages. On admission, physical examination revealed jaundice, hepatomegaly, and pale stools, without splenomegaly. Blood tests showed cholestasis with elevated liver enzymes. At that time, the patient started treatment with ursodeoxycholic acid suspension (15 mg/kg/day, divided into two doses), along with supplementation of fat-soluble vitamins and MCT-enriched formula milk. The hypocholic stools and hypoplastic gallbladder raised suspicion of biliary atresia, but a percutaneous transhepatic cholangiography at 30 days confirmed contrast passage into the intestine, ruling it out.

Further investigations excluded other potential causes of cholestasis, such as alpha-1 antitrypsin deficiency, hypothyroidism, infections and metabolic disorders. An echocardiogram revealed mild pulmonary branch stenosis, and ophthalmological examination identified posterior embryotoxon. Additionally, renal dysplasia was detected on abdominal ultrasound.

**FIGURE 1:**
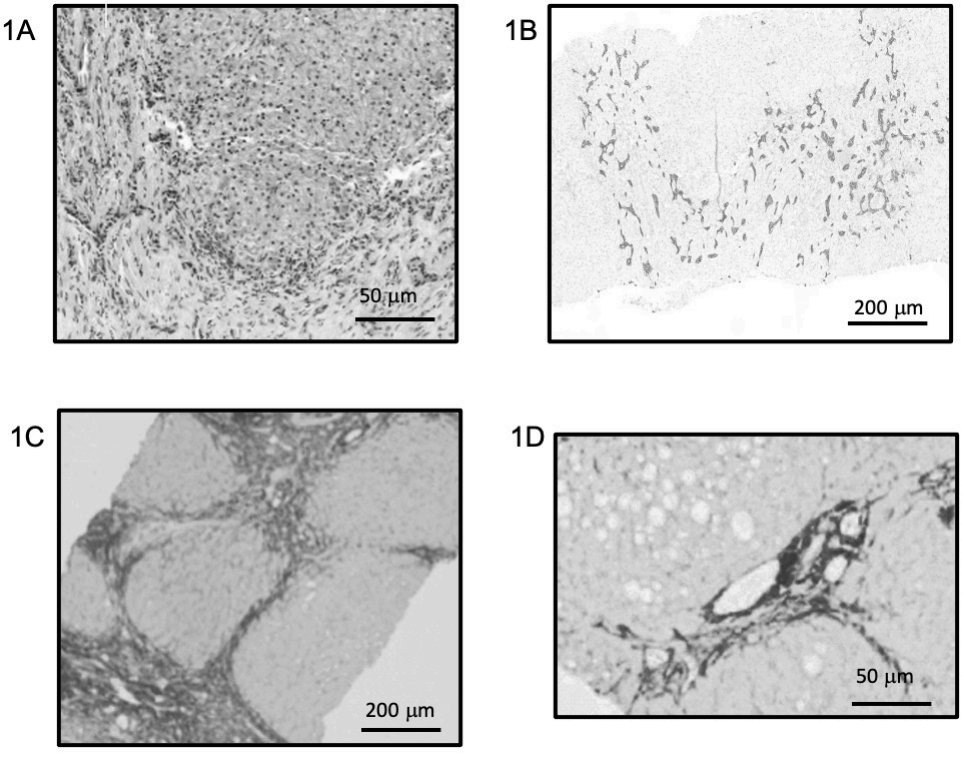
Baseline liver histology and fibrosis evolution after maralixibat treatment. **(A,B)** Hematoxylin and eosin staining **(A)** and cytokeratin 7 immunostaining **(B)** from the initial liver biopsy showing marked intrahepatic cholestasis, disorganization of the hepatic parenchyma, and aberrant ductular proliferation. **(C,D)** Sirius Red staining highlighting fibrosis in the initial biopsy **(C)**, corresponding to Ishak stage 4–5, and clear regression of fibrosis in the follow-up biopsy performed after 1 year of maralixibat treatment **(D)**, with a downgraded stage of F2. Both biopsies were obtained percutaneously from the right hepatic lobe. **(A,D)** ×40 magnification; **(B,C)** ×10 magnification. Staining intensity shown as acquired, with no digital enhancement.

**FIGURE 2:**
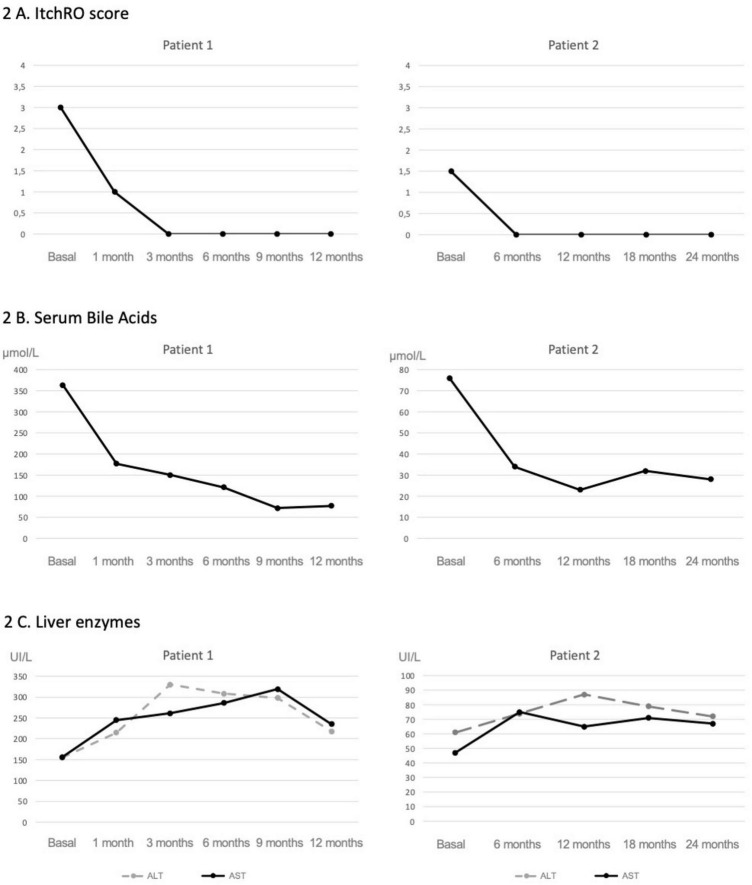
Evolution of pruritus, serum bile acids, and liver enzymes following maralixibat treatment. In patient 1; a rapid and sustained reduction in pruritus was observed shortly after starting maralixibat **(A)**, which occurred in parallel with a marked decline in serum bile acid levels **(B)**. A moderate elevation in transaminases was noted early after treatment initiation, followed by stabilization over time **(C)**. In patient 2, both pruritus and sBA levels **(A,B)** were lower at baseline, and following initiation of maralixibat, they showed a less pronounced but sustained decrease. No remarkable changes were observed in transaminase levels **(C)**.

Exome sequencing at 42 days of life revealed a likely pathogenic variant in the JAG1 gene (NM_000214.3:c.820G > T, p.Gly274Cys; chr20-10633182 C > A) confirming a diagnosis of ALGS. At two and a half months of age, the infant developed irritability and sleep disturbances, attributed to moderate-to-severe pruritus, with ItchRO scores of 2.5 in the morning and 3.0 in the evening (6). For the assessment of pruritus, the mean ItchRO score was calculated over the morning and evening assessments conducted during the 7 days preceding the visit. A nasogastric tube was inserted at 2 months to assist with feeding, and by 7 months, a gastrostomy was required. During this procedure, a liver biopsy was performed revealing intrahepatic cholestasis, aberrant biliary duct proliferation (Figures 1A, B), and cirrhosis (Figure 1C). By this time, the infant also began showing signs of portal hypertension, including splenomegaly (16 cm) and thrombocytopenia (platelet count of 73,000 × 10^9^/L). However, there was no evidence of impaired synthetic or detoxification liver function, with an INR of 0.9, serum albumin of 3.8 g/dL, and ammonia level of 18 μmol/L. Due to the severe pruritus and signs of portal hypertension, the patient was referred for LT evaluation. Before being listed for transplant, the patient joined an early access maralixibat (Livmarli^®^, Mirum Pharmaceuticals, Inc.) program, starting treatment at an oral dose of 190 μg/kg once daily for 1 week to assess tolerability, which was then increased to 380 μg/kg per day. After starting maralixibat, significant improvements were observed in bilirubin levels, total cholesterol, sBA, and pruritus. Liver function and pruritus progression are documented in Figure 2. Thirteen months later, a follow-up liver biopsy performed during a cardiac catheterization revealed a regression of fibrosis to stage F2 (Figure 1D). Both liver biopsy samples were obtained from the right hepatic lobe via the mid-axillary line under ultrasound guidance.

**FIGURE 3:**
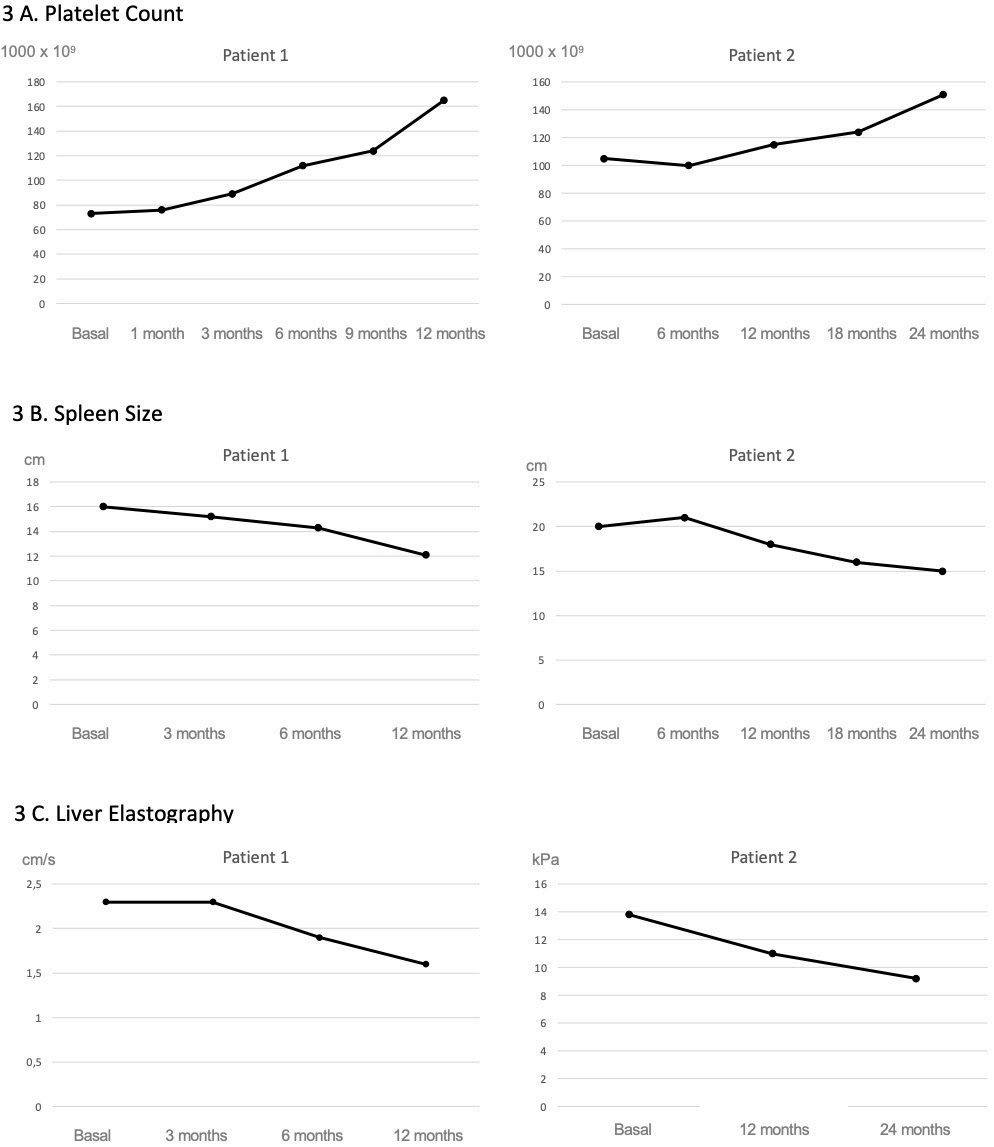
Evolution of platelet count, spleen size and liver elastography following maralixibat treatment. Both patients showed a progressive improvement in platelet counts **(A)** as well as in splenic size assessed by ultrasound **(B)**. In Patient 1, this was accompanied by an improvement in liver stiffness measured by shear wave elastography, whereas in Patient 2, the improvement was observed using transient elastography (FibroScan^®^, Echosens, Paris) **(C)**.

As shown in Figure 2, following initiation of maralixibat and despite the clinical improvements observed, liver enzyme levels increased to approximately twice the baseline values. This prompted an evaluation for alternative causes of hepatocellular injury. A complementary workup was undertaken, including screening for celiac disease, PCR and serological testing for intercurrent viral infections, and a nasopharyngeal swab for respiratory viruses, all of which were negative. Concomitant medications were also reviewed to assess their potential hepatotoxicity; however, aside from maralixibat, the patient was only receiving ursodeoxycholic acid and fat-soluble vitamin supplementation, with serum vitamin levels within the normal range, making a medication-related mechanism other than maralixibat unlikely. As transient elevations in transaminases have been previously reported with maralixibat, and given the marked improvement in pruritus and the stability of synthetic liver function, the decision was made to continue treatment at the same dose without modification. Concurrently, shear wave elastography showed a marked improvement, with liver stiffness decreasing from 2.3 to 1.6 m/s. These findings were consistent with imaging results, which demonstrated a reduction in spleen size from 16 to 12 cm, and a notable increase in platelet count, from 73,000 to 165,000 × 10^9^/L (Figure 3).

Case 2: the second case was a 15-years-old female patient referred from another center with a diagnosis of ALGS, genetically confirmed by exome sequencing at 6 months of life, which revealed a pathogenic variant in the JAG1 gene (NM_000214.2:c.2122_2125del). In addition to the characteristic facial features, the patient presented with branch pulmonary artery stenosis, under cardiology follow-up with favorable evolution. She also showed moderate growth retardation, posterior embryotoxon, and bilateral renal dysplasia without evidence of functional impairment or electrolyte disturbances. No significant pruritus had been previously reported.

At initial evaluation in our center, physical examination revealed a cardiac murmur attributable to the pulmonary stenosis, typical ALGS facial features, splenomegaly measuring 20 cm, and mild excoriation lesions on the forearms. The mean ItchRO score over the 7 days preceding the visit was 1.5 in both the morning and evening assessments, corresponding to mild-to-moderate pruritus. Rifampicin was initiated at a dose of 5 mg/kg every 12 h for the treatment of pruritus in pediatric patients, along with ursodeoxycholic acid (15 mg/kg/day, divided into two doses).

Initial laboratory findings showed hemoglobin of 10 g/dL, leukocyte count of 4,300 × 10^9^/L, and thrombocytopenia with 105,000 × 10^9^/L. Liver enzymes were mildly elevated (ALT 61 U/L and AST 47 U/L), with normal bilirubin levels and serum bile acids at 76 μmol/L.

Abdominal ultrasound demonstrated a nodular liver contour, reduced portal vein velocity, splenomegaly, and a possible splenorenal shunt, findings that were confirmed by contrast-enhanced CT angiography. Transient elastography (FibroScan^®^; Echosens, Paris, France) showed a liver stiffness of 13.8 kPa. Upper gastrointestinal endoscopy revealed grade II gastroesophageal varices, which were not banded.

Due to the presence of pruritus and clinical signs of liver disease with portal hypertension, the patient was started on maralixibat at an initial oral dose of 190 μg/kg once daily for 1 week to assess tolerability, after which the dose was increased to 380 μg/kg per day.

After 24 months of therapy, the patient reported complete resolution of pruritus (ItchRO score 0). Portal hypertension improved, as indicated by a reduction in splenomegaly to 15 cm, a rise in platelet count to 152,000 × 10^9^/L, and a decrease in liver stiffness to 9.2 kPa. Follow-up endoscopy at 12 months showed grade I–II varices.

Liver enzymes remained mildly elevated (ALT 72 U/L and AST 67 U/L), but this did not necessitate any dose adjustment. Serum bile acids decreased to 28 μmol/L (Figure 2).

## Discussion

We report two cases of ALGS treated with maralixibat, which demonstrated improvement not only in pruritus and sBA but also in features of portal hypertension. Bile acids are essential for physiological processes, yet their accumulation is highly toxic to hepatocytes due to their detergent-like properties (7). In ALGS, genetic defects in *JAG1* or *NOTCH2* disrupt the NOTCH signaling pathway, impairing intrahepatic bile duct development (2). This defect leads to intrahepatic bile acid accumulation. Intrahepatic cholestasis, together with the subsequent spill-over of retained constituents into the systemic circulation, may contribute to pruritus, while their accumulation within hepatocytes can induce injury through several mechanisms (8). They destabilize hepatocyte membranes, impair mitochondrial function, and promote reactive oxygen species generation, leading to oxidative injury to proteins, lipids, and DNA (9). This mitochondrial stress may trigger permeability transition, cytochrome c release, and activation of cell death pathways (10). In addition, they induce inflammatory signaling by upregulating cytokines and adhesion molecules such as CCL2, CXCL2, and ICAM-1 through MAP kinase– and early growth response 1–dependent pathways, even in the absence of overt cell death (11). Recent data implicate the methylation-controlled J protein–an inhibitor of mitochondrial respiration–whose increased expression in chronic cholestasis appears to contribute to hepatocellular injury (12). The resulting inflammation drives hepatic fibrosis, cirrhosis, and ultimately portal hypertension. Several clinical trials have demonstrated that IBATi can reduce the reabsorption of BA in the terminal ileum, significantly contributing to a decrease in sBA (3, 4). Although BA levels in hepatocytes have not been directly measured, it is reasonable to assume that there would be a reduction in the accumulation of BA in liver tissue. Consequently, this would decrease the toxic effects of BA on hepatocytes, leading to reduced cell death and, by extension, a lower occurrence of fibrosis in these patients.

In case 1, the patient was assessed for inclusion on the waiting list for LT due to intractable pruritus and significant biochemical abnormalities. Traditionally, patients with severe fibrosis or cirrhosis and unresponsive pruritus would have been clear candidates for LT. However, because liver function was preserved, we initiated a therapeutic trial with maralixibat while simultaneously proceeding with the LT evaluation, thereby maintaining the option of transplantation if clinical improvement proved insufficient.

With the emergence of IBATi such as maralixibat, which have demonstrated rapid pruritus relief in many patients, this approach allowed us to evaluate both symptom control and potential hepatic benefits prior to committing to LT. In this case, the availability of two liver biopsies, performed during unrelated procedures, provided additional insight into maralixibat’s effects, showing improvements not only in clinical symptoms and biochemical markers but also in histological progression. Although liver biopsies are subject to sampling variability, these findings suggest a potential broader therapeutic role for maralixibat.

In this patient, an increase in liver enzymes was observed after maralixibat initiation. Clinical trials have reported temporary reduction or discontinuation of therapy in similar situations. However, given the clear clinical benefit, preserved synthetic function, and overall stability of the patient, treatment was continued without dose modification. The mechanism underlying this transaminase elevation remains unclear. While a direct hepatotoxic effect of the drug has been hypothesized, a more plausible explanation is that impaired bile acid reabsorption forces hepatocytes to increase *de novo* bile acid synthesis, a highly energy-consuming process that may impose metabolic stress on hepatocytes and lead to transient enzyme elevations.

In the second case, the patient presented with significantly lower sBA levels and a more insidious disease course, characterized by mild to moderate pruritus and progressive signs of portal hypertension. Although liver biopsies were not available, improvements observed in transient elastography, imaging studies, and laboratory parameters suggest a reduction in portal hypertension that could be related to a more moderate decrease in sBA levels compared to the first case.

Although total sBA levels were evaluated, the specific bile acid species were not available for analysis in our laboratory. This represents an important limitation of the study, as different bile acid subspecies may have distinct biological activities and could be differentially associated with pruritus severity or hepatic injury. Previous trials have shown that approximately 30% of patients experience pruritus relief without a corresponding reduction in total sBA levels, and that the correlation between sBA and pruritus is generally weak (4). Therefore, any mechanistic interpretations regarding bile acid–mediated effects on pruritus or fibrosis remain speculative. The mechanisms underlying this dissociation remain poorly understood and warrant further investigation. Future studies including detailed bile acid profiling may help to clarify the pathophysiological basis of this observation and identify specific bile acid signatures linked to pruritus.

This study has several inherent limitations. In Case 1, the histological assessment is based on two liver biopsies obtained during unrelated procedures, which may introduce sampling bias and limit the generalizability of the observed improvement in fibrosis. In Case 2, liver biopsies were not available, so hepatic changes were evaluated using non-invasive measures such as transient elastography, imaging, and laboratory parameters. Spontaneous improvement, sampling variability, or natural fluctuation in disease severity remain plausible explanations.

Additional limitations include the small sample size, absence of a control group, and potential confounding factors such as concomitant medications (e.g., ursodeoxycholic acid, rifampicin, vitamin supplementation) and individual variability in disease progression. The lack of detailed bile acid species profiling in both cases also limits the ability to correlate specific bile acid changes with pruritus relief or histological improvement.

These limitations underscore the need for cautious interpretation of the results and highlight the importance of larger, prospective studies to confirm the hepatic benefits of maralixibat in ALGS.

## Conclusion

In our experience with two patients, maralixibat treatment was associated with marked improvement in pruritus and coincided with apparent regression of fibrosis and portal hypertension. However, these observations should be interpreted with caution due to the small sample size, uncontrolled design, and other limitations such as variable biopsy timing, concomitant medications, and lack of detailed bile acid profiling. These preliminary findings suggest a possible association but do not allow conclusions about causality, highlighting the need for further investigation in larger, prospective studies.

## Data Availability

The original contributions presented in this study are included in this article/supplementary material, further inquiries can be directed to the corresponding author.

## References

[1] Vandriel S Li L She H Wang J Gilbert M Jankowska I Natural history of liver disease in a large international cohort of children with Alagille syndrome: results from the GALA study. *Hepatology.* (2023) 77:512–29. 10.1002/hep.32761 36036223 PMC9869940

[2] Gilbert M Bauer R Rajagopalan R Grochowski C Chao G McEldrew D Alagille syndrome mutation update: comprehensive overview of JAG1 and NOTCH2 mutation frequencies and insight into missense variant classification. *Hum Mutat.* (2019) 40:2197–220. 10.1002/humu.23879 31343788 PMC6899717

[3] Ovchinsky N Aumar M Baker A Baumann U Bufler P Cananzi M Efficacy and safety of odevixibat in patients with Alagille syndrome (ASSERT): a phase 3, double-blind, randomised, placebo-controlled trial. *Lancet Gastroenterol Hepatol.* (2024) 9:632–45. 10.1016/S2468-1253(24)00074-8 38670135

[4] Gonzales E Hardikar W Stormon M Baker A Hierro L Gliwicz D Efficacy and safety of maralixibat treatment in patients with Alagille syndrome and cholestatic pruritus (ICONIC): a randomised phase 2 study. *Lancet.* (2021) 398:1581–92. 10.1016/S0140-6736(21)01256-3 34755627

[5] Hansen B Vandriel S Vig P Garner W Mogul D Loomes K Event-free survival of maralixibat-treated patients with Alagille syndrome compared to a real-world cohort from GALA. *Hepatology.* (2024) 79:1279–92. 10.1097/HEP.0000000000000727 38146932 PMC11095900

[6] Kamath B Abetz-Webb L Kennedy C Hepburn B Gauthier M Johnson N Development of a novel tool to assess the impact of itching in pediatric cholestasis. *Patient.* (2018) 11:69–82. 10.1007/s40271-017-0266-4 28710680 PMC5766715

[7] Chiang J. Recent advances in understanding bile acid homeostasis. *F1000Research.* (2017) 6:2029. 10.12688/f1000research.12449.1 29188025 PMC5698910

[8] Kamath B Baker A Houwen R Todorova L Kerkar N. Systematic review: the epidemiology, natural history, and burden of Alagille syndrome. *J Pediatr Gastroenterol Nutr.* (2018) 67:148–56. 10.1097/MPG.0000000000001958 29543694 PMC6110620

[9] Li M Cai S Boyer J. Mechanisms of bile acid mediated inflammation in the liver. *Mol Aspects Med.* (2017) 56:45–53. 10.1016/j.mam.2017.06.001 28606651 PMC5662014

[10] Yerushalmi B Dahl R Devereaux M Gumpricht E Sokol R. Bile acid-induced rat hepatocyte apoptosis is inhibited by antioxidants and blockers of the mitochondrial permeability transition. *Hepatology.* (2001) 33:616–26. 10.1053/jhep.2001.22702 11230742

[11] Allen K Jaeschke H Copple B. Bile acids induce inflammatory genes in hepatocytes: a novel mechanism of inflammation during obstructive cholestasis. *Am J Pathol.* (2011) 178:175–86. 10.1016/j.ajpath.2010.11.026 21224055 PMC3070591

[12] Iruzubieta P Goikoetxea-Usandizaga N Barbier-Torres L Serrano-Maciá M Fernández-Ramos D Boosting mitochondria activity by silencing MCJ overcomes cholestasis-induced liver injury. *JHEP Rep.* (2021) 3:100276. 10.1016/j.jhepr.2021.100276 33997750 PMC8099785

